# Discoidin domain receptor 2 activation of p38 mitogen-activated protein kinase as an important pathway for osteonectin-regulating osteoblast mineralization

**DOI:** 10.1186/s13018-021-02860-1

**Published:** 2021-12-07

**Authors:** Yun-Sen Zhu, Jiang-Nan Zhang, Ting-Ting Mo, Chang Jiang, Ru-Chao Ma, Liang Chen

**Affiliations:** 1 Department of Orthopaedic Surgery, The First People’s Hospital of Wenling, Chuan’an Nan Road NO 333, Wenling, 317500 Zhejiang China; 2 Department of Orthopaedic Surgery, The First Affiliated Hospital of Soochow University, Suzhou, 215000 Jiangsu China

**Keywords:** Osteonectin, p38 MAPK signaling pathway, Discoidin domain receptor 2, Mineralization

## Abstract

**Objective:**

The present study aimed to determine the role of the discoidin domain receptor 2 (DDR2) in the osteonectin (ON) regulation of osteoblast mineralization through the activation of p38 mitogen-activated protein kinase (MAPK).

**Methods:**

Four groups were established: the ON group, the inhibitor group, the *Ddr2*-small interfering ribonucleic acid (siRNA) group, and the control group. Osteoblasts from the parietal bones of neonatal Sprague–Dawley rats were isolated and cultured. In the ON group, 1 µg/mL ON was added to the osteoblasts. The gene expressions of collagen 1 (*Col 1*) and *Ddr2* were detected using reverse transcription-quantitative polymerase chain reaction (RT-qPCR). In the inhibitor group, the osteoblasts were added to WRG-28 (a specific DDR2 inhibitor), and in the *Ddr2*-siRNA group, the osteoblasts were transfected with *Ddr2*-siRNA. The gene and protein expressions of DDR2, bone sialoprotein, osteocalcin, osteopontin, and p38 MAPK were determined using RT-qPCR and western blot analysis. Alizarin red staining and transmission electron microscopy were used to detect mineralization.

**Results:**

The results showed that ON enhanced the osteoblast *Col 1* and *Ddr2* gene expressions, while the use of a *Ddr2*-siRNA/DDR2-blocker decreased the OPN, BSP, OCN, and P38 gene and protein expressions and reduced osteoblast cellular activity and mineralized nodules.

**Conclusion:**

The present study demonstrated that DDR2 activation of p38 MAPK is an important approach to ON-regulating osteoblast mineralization.

## Introduction

Osteonectin (ON) is an important non-collagenous protein, also known as SPARC (secreted protein acidic and rich in cysteine) or BM‐40, and it has a high affinity for binding with collagen and hydroxylapatite [1]; ON also participates in the overall osteoblast mineralization process [2] and plays an important role as a type of functional regulatory protein in the function of osteoblasts and the synthesis of collagen and extracellular matrices [3–7]. An important signaling pathway, regarded as a signal regulatory hub in bone repair and reconstruction, is p38 mitogen-activated protein kinase (MAPK) [8–11]. Previous studies of Zhu et al. verify the importance of P38 for ON regulation in osteoblast mineralization [12]. Although P38 pathway activation using ON has been fully proven as a potential mechanism in various other cellular physiological and pathological processes [13–15], this signal event was first reported in osteoblast mineralization; however, its specific mechanism remains unclear.

Domain receptors (DDRs) on cell surfaces are widely expressed tyrosine kinase receptors; they can be slowly and continuously activated by triple-helix collagen [16]. Triple-helix collagen peptides contain crystalline structures of discoid protein domains, which can lead to structural changes and direct phosphorylation of DDR2. [17]. Existing studies have comprehensively demonstrated that ON regulates the growth and deposition of collagen fibers and can stimulate collagen conformational changes [2]. In addition, the fact that DDR2 and ON share the same collagen recognition pattern and combine the same GVMGFO motif suggests a correlation between the two [18, 19]. Their association has also been illustrated in experimental studies, where DDR2 phosphorylation promoted fibroblast responses to tissue regeneration and healing, while DDR2 deletion seriously delayed wound healing; accordingly, ON expression was isotropically reduced [20].

The DDR2 tyrosine kinase domain is considered a key signal transmission site for P38 activation in many cell activities. The DDR-initiated downstream p38 MAPK pathway is the major mechanism for matrix metalloproteinase regulation [21]. Many transforming growth factors can adjust the release of calcified extracellular vesicles and affect collagen deposition in vascular smooth muscle cells through the activation of p38 MAPK via DDRs [22, 23]. DDR2 is also considered to be a key regulator of osteoblast differentiation, and its stimulation of p38 MAPK is a necessary condition for DDR2-induced activation of RUNX2 and OCN promoters [24]. The purpose of this study is to investigate whether DDR2 is also an important pathway for ON activation of P38 in the regulation of osteoblast mineralization.

## Materials and methods

### Primary osteoblast isolation, culturing, and purification

As previously described, osteoblasts were isolated from the parietal bones of neonatal (aged < 48 h) Sprague–Dawley rats through sequential collagenase [25]. The released cells were collected by centrifugation (1000 rpm for 10 min), washed with phosphate‐buffered saline (PBS) (GNM-20012, China) twice, suspended in an α‐minimum essential medium (α‐MEM) (4150034, Gibco), seeded at a density of 5 × 10^5^ cm^−2^, and incubated at 37 °C in a humidified 5% CO_2_ atmosphere. When the cells reached a confluence of approximately 80–90%, they were purified using a different attachment method with the aim to remove the mixture of fibroblasts and endothelial cells, both of which were relatively rapid adherents, so that most of the two cell types remained in the previous dish for purification after a transfer every 10 min. Following the identification with alkaline phosphatase (ALP) staining and alizarin red staining (ARS), the osteoblasts were cultured for subsequent experiments.

### *Ddr2*-siRNA construction and transfection

The following small interfering ribonucleic acid (siRNA) sequences were used: 5′-AACCTGATGACCTGAA GGAGT-3′ (NM_006182, base pairs 621–641). The cells (osteoblasts) were cultured in T150 (Corning) culture flasks in a Roswell Park Memorial Institute (RPMI) 1640 (R7509-1L, SIGMA) medium and 10% fetal bovine serum (FBS) (A31608-02, Gibco) without antibiotics until a confluence of 80% was reached. The cells were rinsed twice with PBS, and siRNA and 5% CO_2_ were added for 5 h at 37 °C. It was then replaced with a fresh RPMI 1640 medium containing 10% FBS, and the cells were further incubated for 72 h before being used in specific experiments. The following items were prepared for siRNA transfection: (1) tube 1: 61 µL of siRNA + 2.2 mL of RPMI 1640; and (2) tube 2: 90 µL of Lipofectamine 2000 (Lipo 2000, no. 11668019; Invitrogen Company) + 2.16 mL of RPMI 1640. The tubes were incubated for 5 min at room temperature, then combined and further incubated for 20 min. Next, 13.5 mL of RPMI 1640 medium was added; the siRNA mix was then applied to the cells. The transfection efficiency was assessed using a fluorescence microscope.

### Cell grouping and induced mineralization

The second-generation osteoblasts were re-inoculated in a six‐well plate at a density of 2 × 10^5^ cells/mL; they were then cultured in α‐MEM with 10% FBS. After mixing the cells, the original medium was aspirated, and the cells were randomly assigned to different groups and studied in three stages.

#### Stage one

In the control group, the osteoblasts were added to a mineralized solution containing 50 mg/L ascorbic acid, 10 mmol/L sodium glycerophosphate, and a 100 mL/L FBS α‐MEM culture solution. In the ON group, the osteoblasts were added to a mineralized solution + 1 µg/mL ON (Sino Biological 8087-R08H). The medium was substituted every 2 days in both groups. On day 5, the *Col1 a1*, *Col1 a2*, and *Ddr2* gene expressions were detected using reverse transcription-quantitative polymerase chain reaction (RT-qPCR) (according to the previous study [12]). The experiments were conducted in triplicates.

#### Stage two

In the control group, the osteoblasts were added to the mineralized solution; in the ON group, the osteoblasts were added to a mineralized solution + 1 µg/mL ON; and in the inhibitor group, the osteoblasts were added to a mineralized solution + 1 µg/mL ON + 230 nM WRG-28 (a DDR2-specific inhibitor, CAS No. 1913291-02-7, ChemeGen) [26]. The medium was substituted every 2 days in each group. On day 5, the non-collagen protein (OPN, BSP, OCN), *Ddr2*, and *P38* gene expressions were detected using RT-qPCR. The experiments were conducted in triplicates.

#### Stage three

In the control group, the osteoblasts were added to a mineralized solution; in the ON group, the osteoblasts were added to a mineralized solution + 1 µg/mL ON; in the inhibitor group, the osteoblasts were added to a mineralized solution + 1 µg/mL ON + 230 nM WRG-28; and in the DDR2-siRNA group, the osteoblasts were transfected with DDR2-siRNA and added to a mineralized solution + 1 µg/mL ON. The medium was substituted every 2 days in each group. On day 5, the DDR2, p38, OPN, BSP, and OCN gene and protein expressions were determined using RT-qPCR and western blot. The experiments were conducted in triplicates. The mineralized nodules were stained using ARS, and the cell ultrastructure was observed using transmission electron microscopy (TEM) (HITACHI HT7700).

### RT-qPCR

Cells were collected from each group, and the total ribonucleic acid (RNA) was extracted using a Trizol reagent (no. 15596026; Ambion Company) in accordance with the manufacturer’s protocols. Next, 2 µg of total RNA was used for reverse transcription, and an RT‐PCR was performed using an RT‐PCR kit (no. RR037A; Takara Company) in accordance with the manufacturer’s instructions. The *Col1 a1*, *Col1 a 2*, *Ddr2*, *P38*, *Opn*, *Bsp*, and *Ocn* gene expression levels were analyzed using the Light Cycler® 96 real‐time PCR system (Roche), with glyceraldehyde 3-phosphate dehydrogenase (GAPDH) as an internal control gene. The primer sequences are listed in Table 1. All real‐time PCRs were performed in triplicate, and the results after calibration with GAPDH expression were calculated using the ΔΔCT method; they are presented in fold increase and relative to the control.

**Table 1:** Primer sequences for RT‐qPCR

Target gene	Primer sequence (5′–3′)
Mouse COL1 a1	F: GACGCCATCAAGGTCTACTG
	R: ACGGGAATCCATCGGTCA
Mouse COL1 a2	F: GGAGGGAACGGTCCACGAT
	R: GAGTCCGCGTATCCACAA
Mouse DDR2	F: CTCCCAGAATTTGCTCCAG
	R: GCCACATCTTTTCCTGAGA
Mouse P38	F: GGATATTTGGTCCGTGGGCT
	R: CCGTCAGACGCATTATCTGC
Mouse OPN	F: CCAGCCAAGGACCAACTACA
	R: AGTGTTTGCTGTAATGCGCC
Mouse BSP	F: AGAAAGAGCAGCACGGTTGA
	R: AATCCTGACCCTCGTAGCCT
Mouse OCN	F: ATTGTGACGAGCTAGCGGAC
	R: TCGAGTCCTGGAGAGTAGCC
Mouse GAPDH	F: CCTGCACCACCAACTGCTTA
	R: CATCACGCCACAGCTTTCCA

*COL1* Collagen 1; *DDR2* Disc protein domain receptor 2; *P38* P38 mitogen‐activated protein kinase; *BSP* bone sialoprotein; *OCN* osteocalcin; *F* forward; *R* reverse; *RT‐qPCR* reverse transcription-quantitative polymerase chain reaction

### Western blot analysis

The proteins extracted from osteoblasts were quantified using a bicinchoninic acid assay protein assay kit (Beyotime) in accordance with the manufacturer’s instructions. The cells cultured in the six-well plate were washed with PBS three times, and an appropriate amount of RIPA lysate (Beyotime) was added to phenylmethylsulfonyl fluoride (PMSF) (Amersco-0754-100G) within a few minutes before use (the sinal PMSF concentration was 1 mM). Then, 200 µL of pyrolysate was added to each hole and mixed well; after full decomposition and 10,000 × *g* centrifuging for 5 min, the supernatant was taken. A suitable amount of BCA working fluid (P0011, Beyotime) was prepared by adding 50 volumes of BCA reagent A and 1 volume of BCA reagent B (50:1), the appropriate volume of the sample was added to a 1.5 mL centrifuge tube and supplemented with a 0.9% NaCL solution to 100 µL. Next, 1 mL of BCA working fluid was added to each hole and left at 37 °C for 30 min; the A562 absorption value was then determined and the protein concentration calculated according to the standard curve. The protein of each group was loaded on sodium dodecyl sulfate‐polyacrylamide gel electrophoresis and transferred onto nitrocellulose membranes. The membranes were blocked with 5% skim milk in Tris‐buffered saline with Tween 20 at room temperature for 1 h. They were then supplemented with the primary antibody (1:1000) and incubated at 4 ℃ for 12 h. The membranes were then incubated with a secondary antibody (1:2000) at room temperature for 2 h. Protein bands were developed using enhanced chemiluminescence reagents (Millipore), the gel grayscales were captured using the ImageJ (V1.8.0) software, and the relative expression quantity was calculated using the grayscale-to-GAPDH ratio.

### Alkaline phosphatase staining

The original culture medium was removed, and the cells were washed twice using PBS and fixed in 2.5% glutaraldehyde for 24 h. They were then washed three to five times with PBS and stained with an ALP solution (BCIP-NBT, C3206, Beyotime) in the dark for 30 min. Following ALP removal, the cells were washed in distilled water two to three times and observed under a microscope.

### ARS

A volume of 1% alizarin red aqueous solution was obtained by dissolving 1 g of alizarin red powder (A5533, Sigma) in 100 mL of distilled water. Impurities were filtered, the PH adjusted to 4.2 with 10% ammonia, and the solution stored at 4 °C for later use. After a two-week mineralized solution induction, the osteoblasts were washed with PBS and fixed with 95% ethanol for 30 min. After drying, the prepared ARS solution was added for 15 min; the osteoblasts were then rinsed three times with distilled water, dewatered, sealed, and observed and photographed under a light microscope (Olympus) for calcification detection. Adobe Photoshop CS5 was used for image analysis to calculate the image-field percentage of positive calcium nodule staining. The procedure was as follows: (1) the image was opened in Adobe Photoshop CS5 and the layer background copied; (2) a new solid color fill or adjustment layer was created and a bright green background chosen; (3) “blending options” in the first layer were selected and the filter range of each color in the blending ribbon adjusted. R, 120–255, G, 0–112 and B, 0–112 were selected for this analysis; (4) layer 1 was converted to a smart object, the histogram palette opened, and “expanded view” selected; and (5) the positive stain percentage of the calcium nodule was calculated by the number of pixels in the full image and the number of pixels in the layer.

### Cell ultrastructure observation

The osteoblasts were washed with PBS two times, fixed with 2.5% glutaraldehyde, phosphoric acid buffer, and 1% osmium acid solution, respectively, rinsed with 0.1 M phosphoric acid rinsing solution, and dehydrated at 4 °C with ethanol and acetone in a refrigerator. After embedding with pure acetone and an embedding solution, the samples were cured in an oven, sliced to 50–60 nm by ultrathin sectioning machine, and stained with 3% uranyl acetate and lead citrate. The cell ultrastructure was observed using a TEM (HITACHI HT7700).

### Statistical analysis

All data were tested for normality and homogeneity of variance; the measurement data were presented with the mean ± standard deviation (SD). A one‐way analysis of variance was conducted for the comparison among multiple groups, and tests on the least significant difference (Student–Newman–Keuls test or *q* test) were conducted for the comparison between two groups with homogeneity of variance; comparisons between two groups without homogeneity of variance were highlighted using Tamhane’s T2 test. A *P* value of < 0.05 was considered statistically significant. All statistical analyses were performed using the SPSS (17.0) software.

## Results

### ON enhancement of osteoblast *Col1* and *Ddr2* gene expressions

In the first stage of the experiment, the osteoblasts were divided into two groups: the ON group and the control group. On day 5, RT‐qPCR was conducted to measure the mRNA expressions of *Col1 a1*, *Col1 a2*, and *Ddr2*.

**Fig. 1:**
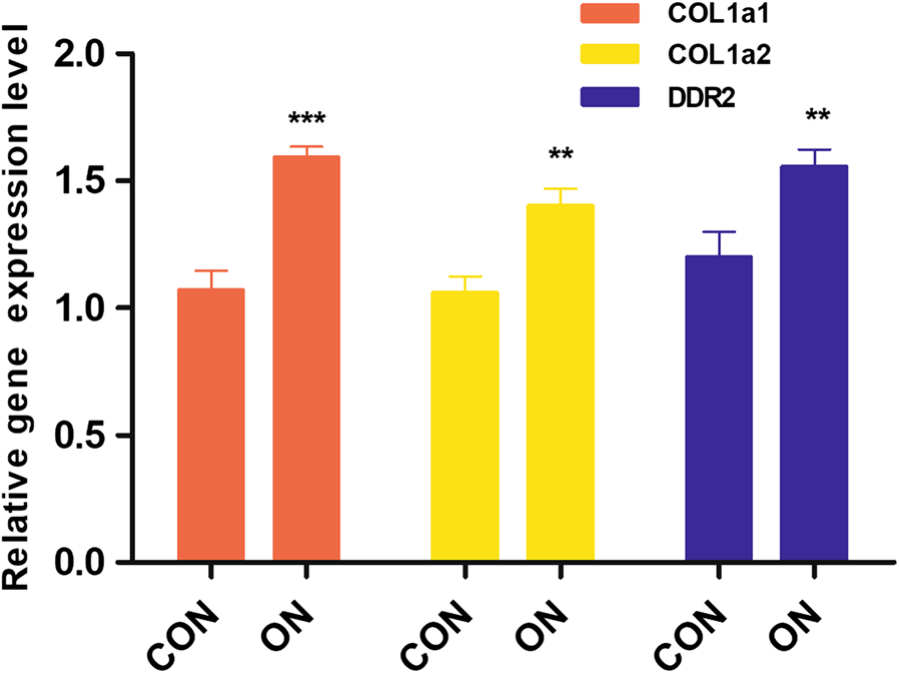
Osteonectin enhanced the gene expressions of COL1 and DDR2. The mRNA expressions of COL1a1, COL1a2 and DDR2 were quantified by RT-qPCR; **P < .01, ***P < .001 versus the CON group; the experiments were conducted in triplicates; data were expressed by means ± standard deviation (SD). COL1: collagen 1; DDR2: discoidin domain receptor 2; RT-qPCR: reverse transcription-quantitative polymerase chain reaction; CON group: control group, the osteoblasts were added with mineralized solution; ON group: osteonectin group, the osteoblasts were added with mineralized solution + 1 µg/mL osteonectin

The results showed that the *Col1 a1*, *Col1 a2*, and *Ddr2* expressions were significantly increased in the ON group compared with the control group (*Col1 a1*, *P* < 0.001; *Col1 a2*, *P* < 0.01; and *Ddr2*, *P* < 0.01) (Fig. 1).

### DDR2-blocker decrease in *Opn*, *Bsp*, *Ocn*, and* P38* gene expressions

In the second stage of the experiment, the inhibitor group was set up and the gene expressions of non-collagen proteins (OPN, BSP, OCN), DDR2, and P38 were detected using RT-qPCR.

**Fig. 2:**
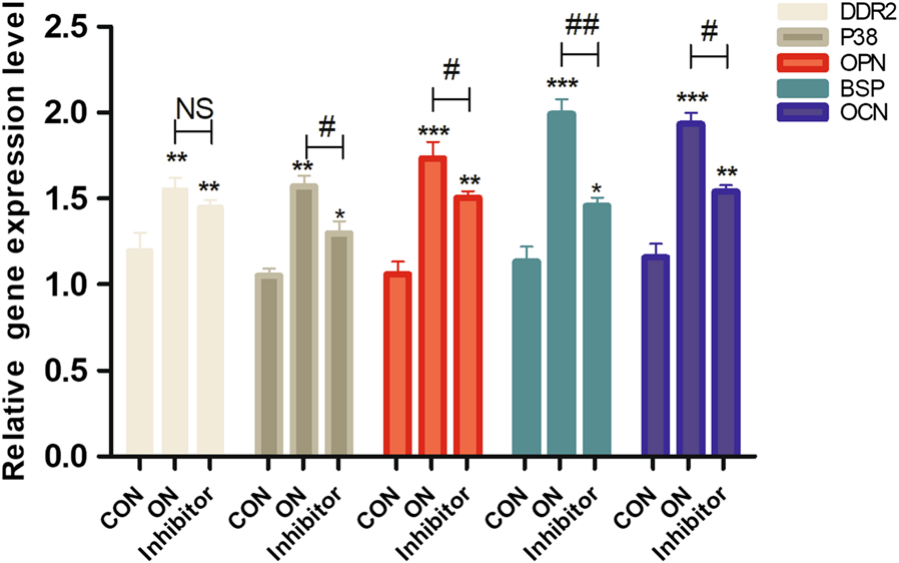
DDR2-blocker decreased the gene expressions of OPN, BSP, OCN and P38. the mRNA expressions of OPN, BSP, OCN, DDR2 and P38 were quantified by RT-qPCR; *P < .05, **P < .01, ***P < .001 versus the CON group; NS *P* > .05, #*P* < .05, ##*P* < .01 versus the ON group; the experiments were conducted in triplicates; data were expressed by means ± standard deviation (SD). OPN, osteopontin; BSP, bone sialoprotein; OCN, osteocalcin; P38: p38 mitogen‐activated protein kinase; DDR2: discoidin domain receptor 2; RT-qPCR: reverse transcription-quantitative polymerase chain reaction; CON group: control group, the osteoblasts were added with mineralized solution; ON group: osteonectin group, the osteoblasts were added with mineralized solution + 1 µg/mL osteonectin; inhibitor group: the osteoblasts were added with mineralized solution + 1 µg/mL osteonectin + 230 nM WRG-28

The ***Opn*, *Bsp*, *Ocn, and P38*** gene expressions were significantly decreased in the inhibitor group compared with the ON group (***Opn***, *P* < 0.05; ***Bsp***, *P* < 0.01; ***Ocn***, *P* < 0.05; and p38, *P* < 0.05); there was no statistical difference in DDR2 gene expression between the two groups (*P* > 0.05). The ***Opn*, *Bsp*, *Ocn, and P38*** gene expressions were still increased in the inhibitor group compared with the control group; the differences were all statistically significant (***Opn***, *P* < 0.01; ***Bsp***, *P* < 0.05; ***Ocn***, *P* < 0.01; and ***P38***, *P* < 0.05) (Fig. 2).

### DDR2-siRNA/DDR2-blocker down-regulation of positive ON regulatory effect on osteoblasts

In the third stage of the experiment, *Ddr2*-siRNA was constructed to suppress DDR2 expression in osteoblasts. The DDR2, p38, OPN, BSP, and OCN gene and protein expressions were determined using RT-qPCR and western blot. The mineralized nodules were stained using ARS, and the cell ultrastructure was observed via TEM.

**Fig. 3:**
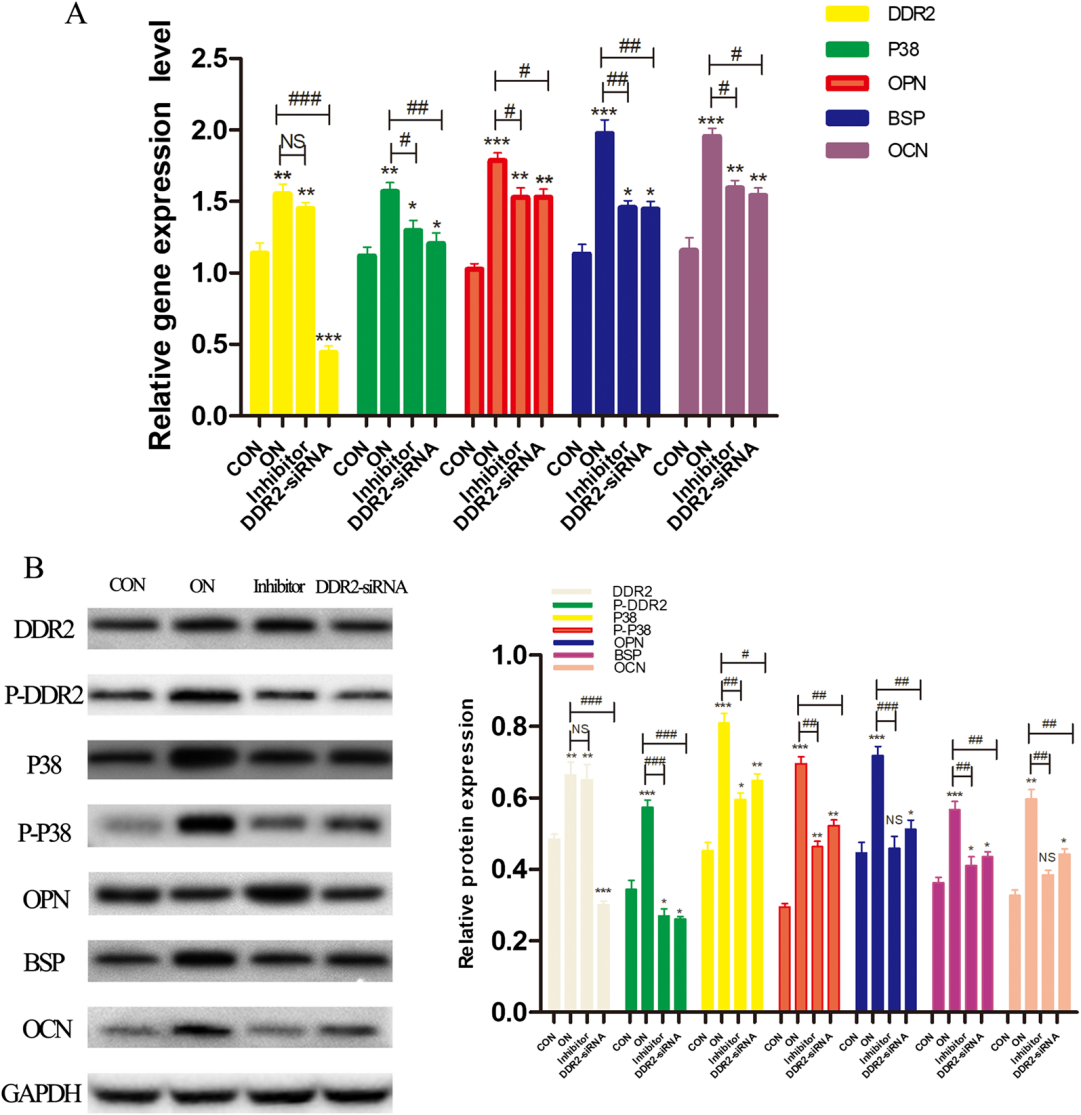
DDR2-siRNA/DDR2-blocker downregulated the gene and protein expressions of OPN, BSP, OCN and P38. **A** the mRNA expressions of OPN, BSP, OCN, DDR2 and P38 quantified by RT-qPCR in response to the treatment of CON, ON, Inhibitor, and DDR2-siRNA group; **B** the gray value of protein bands and protein levels of OPN, BSP, OCN, DDR2, P-DDR2, P38 and P-P38 quantified by western blot analysis in response to the treatment of CON, ON, Inhibitor, and DDR2-siRNA group; NS *P* > .05, *P < .05, **P < .01, ***P < .001 versus the Con group; #*P* < .05, ##*P* < .01, ###*P* < .001 versus the ON group; the experiments were conducted in triplicates; data were expressed by means ±  standard deviation (SD). OPN, osteopontin; BSP, bone sialoprotein; OCN, osteocalcin; P38: p38 mitogen‐activated protein kinase; P-P38: phosphorylated p38; DDR2: discoidin domain receptor 2; P-DDR2: phosphorylated DDR2; RT‐qPCR: reverse transcription-quantitative polymerase chain reaction; CON group: control group, the osteoblasts were added with mineralized solution; ON group: osteonectin group, the osteoblasts were added with mineralized solution + 1 µg/mL osteonectin; Inhibitor group: the osteoblasts were added with mineralized solution + 1 µg/mL osteonectin + 230 nM WRG-28; DDR2-siRNA group: the osteoblasts were transfected with DDR2-siRNA and added with mineralized solution + 1 µg/mL osteonectin

The DDR2 and phosphorylated DDR2 (P-DDR2) gene and protein expressions in the DDR2-siRNA group were observably decreased compared with the ON group (mRNA: *Ddr2*, *P* < 0.001; protein: DDR2, *P* < 0.01; and P-DDR2, *P* < 0.001). The P-DDR2 protein expression in the inhibitor group was also significantly decreased (*P* < 0.001); however, no obvious differences were observed in DDR2 gene and protein expressions between the ON group and the inhibitor group (*P* > 0.05). Correspondingly, the OPN, BSP, OCN, and p38 gene and protein expressions in the inhibitor group and the DDR2-siRNA group were both observably decreased compared with the ON group (*P* < 0.05 in all); however, the expressions were still significantly increased compared with the control group (*P* < 0.05 in all) (Fig. 3).

**Fig. 4:**
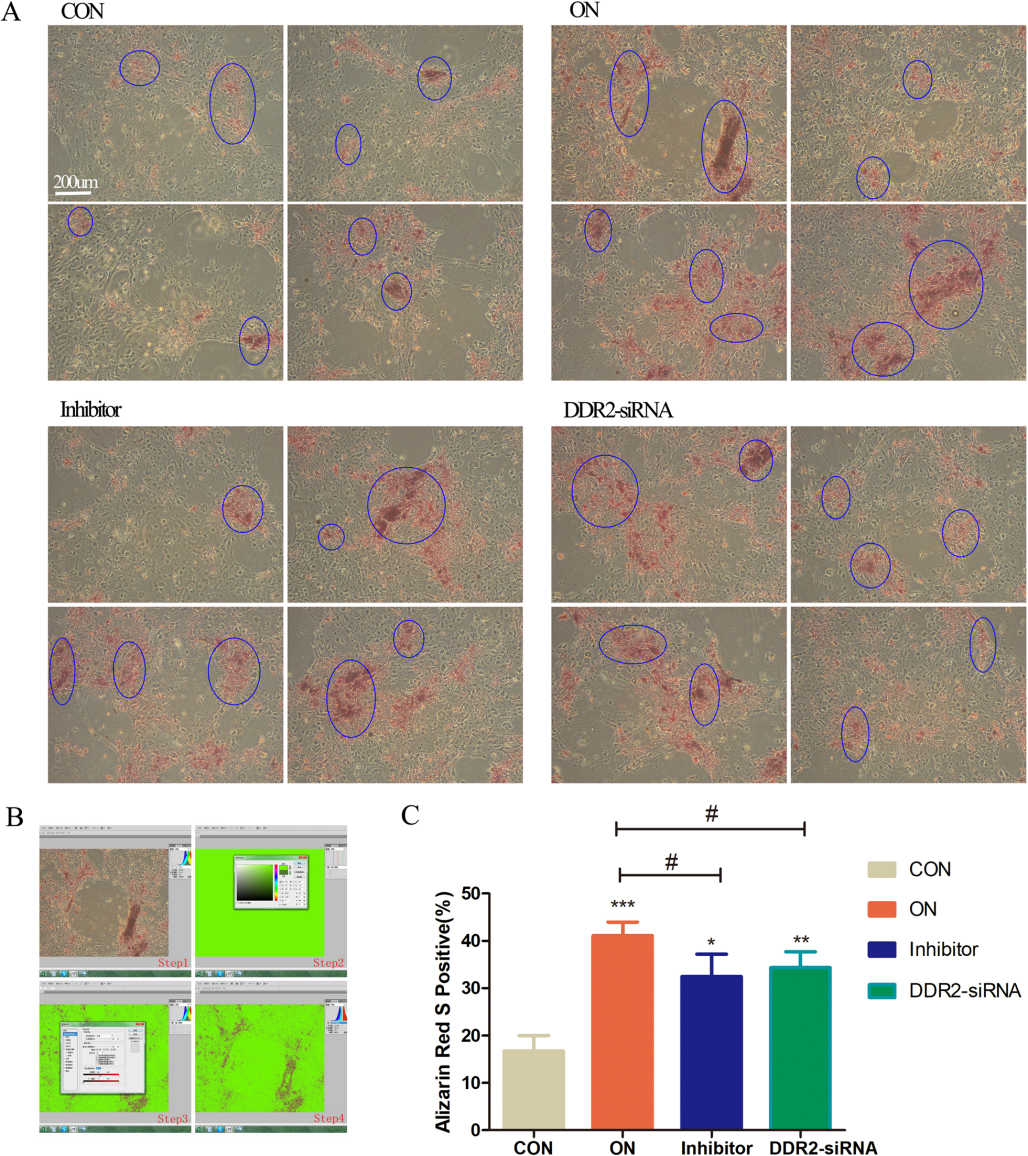
DDR2-siRNA/DDR2-blocker reduced the formation of mineralized nodules. **A** Calcium nodules (blue circle) observation stained with alizarin red under a microscope (× 100) after induced mineralization for 14 days in response to the treatment of CON, ON, Inhibitor and DDR2-siRNA group; **B** the Adobe Photoshop CS5 system was used to calculate the positive staining of calcium nodules; **C** quantitative analysis of calcium nodules in each group. Bars, (a) 200 µm, *P < .05, **P < .01, ***P < .001 versus the Con group; #*P* < .05 versus the ON group; three samples in each group were observed; data were expressed by means ± standard deviation (SD); CON group: control group, the osteoblasts were added with mineralized solution; ON group: osteonectin group, the osteoblasts were added with mineralized solution + 1 µg/mL osteonectin; Inhibitor group: the osteoblasts were added with mineralized solution + 1 µg/mL osteonectin + 230 nM WRG-28; DDR2-siRNA group: the osteoblasts were transfected with DDR2-siRNA and added with mineralized solution + 1 µg/mL osteonectin

The DDR2-siRNA/DDR2-blocker reduced the formation of mineralized nodules. Mineralized nodule areas in the DDR2-siRNA group and the inhibitor group were significantly smaller compared with the ON group but still larger than in the control group (*P* < 0.05 in all) (Fig. 4).

**Fig. 5:**
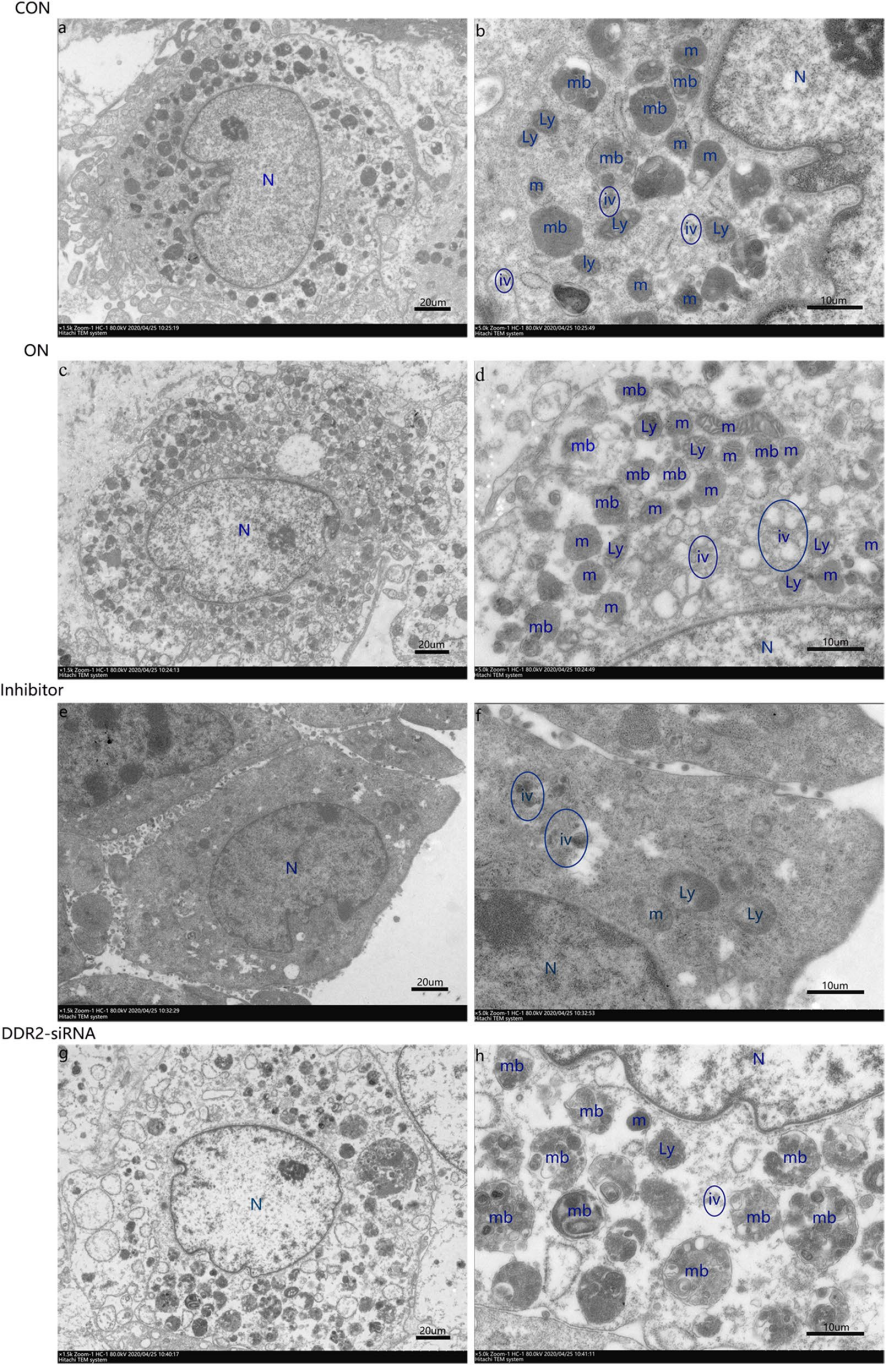
DDR2-siRNA/DDR2-blocker suppressed the osteogenic activity. The cell ultrastructure of osteoblasts was observed by TEM in response to the treatment of CON, ON, Inhibitor, and DDR2-siRNA group; DDR2-siRNA/inhibitor group both showed fewer organelles and intracellular vesicles. Bars: **a**, **c**, **e** and **g**, 20 µm; Bars: **b**, **d**, **f** and **h**, 10 µm; N, nucleus; m, mitochondria; ly, lysosomes; mb, multivesicular bodies; iv, intracellular vesicle; TEM, transmission electron microscopy; CON group: control group, the osteoblasts were added with mineralized solution; ON group: osteonectin group, the osteoblasts were added with mineralized solution + 1 µg/mL osteonectin; Inhibitor group: the osteoblasts were added with mineralized solution + 1 µg/mL osteonectin + 230 nM WRG-28; DDR2-siRNA group: the osteoblasts were transfected with DDR2-siRNA and added with mineralized solution + 1 µg/mL osteonectin

The *Ddr2*-siRNA/DDR2-blocker suppressed osteogenic activity in the osteoblasts. The osteoblasts in the *Ddr2*-siRNA group and the inhibitor group both exhibited low activity, and fewer organelles and vesicles were observed (Fig. 5).

## Discussion

Various cytokines and signaling pathways are involved in osteoblast mineralization. The previous studies showed that ON had a significant positive role in the regulation of osteoblast mineralization through the p38 signaling pathway [12]; however, the exact mechanism of ON activation of p38 is still unknown. ON has a functional and structural basis for regulating a variety of cytokines and binding to various cell surface receptors. As a type of cell surface tyrosine kinase receptor, DDR2 is also actively associated with osteoblast differentiation, maturation, and mineralization. ON has the potential to affect DDR2 expression by regulating collagen synthesis and conformational changes (or in a variety of other ways). In this study, the *Ddr2* expression was detected by adding ON to the osteoblasts; the P38 and mineralization indicator expressions were then investigated by blocking osteoblast DDR2 signaling pathways. The results indicated that DDR2 was an important pathway for ON activation of P38 in the regulation of osteoblast mineralization.

Based on the previous research, 1 µg/mL of ON was initially added to the osteoblasts [12]; the results showed that ON observably elevated *Col1* and *Ddr2* gene expressions. As the collagen chaperone, ON interacted with endoplasmic reticulum molecules to effectively transport non-mutated procollagen molecules out of the endoplasmic reticulum, thus further enhancing collagen synthesis [27]. Osteonectin can also affect collagen diameter and quantity by regulating transglutaminase activity [28]. The binding of ON to collagen and calcium ions is a critical step for the assembly of collagen fibers into bundles. It is suggested that the highly negatively charged molecular structure of ON can further promote collagen fiber assembly by binding to the highly positively charged collagen and calcium ion regions through electrostatic interaction [29]. Moreover, ON affects the processing, deposition, and degradation of procollagen by regulating integrins in order to improve the matrix assembly process [30]. Correspondingly, DDR2 is a collagen-binding receptor tyrosine kinase, and the GVMGFO collagen motif is believed to be the primary site for specific DDR2 recognition and binding; this combination causes a DDR structural change and subsequent phosphorylation and activation, which are extensively involved in cell development, extracellular matrix turnover, growth regulation, and cancer-related functions [31, 32]. Therefore, ON can influence the expression of DDR2 activation by regulating the growth and recombination of collagen; this is in line with the results of the present experiment.

To explore whether activated DDR2 can further activate the P38 signaling pathway, WRG-28 (a specific DDR2 inhibitor) was used to block the DDR2 signaling pathway in the second stage of the experiment. The results showed that the expressions of P38 and non-collagen genes were significantly decreased in the inhibitor group, indicating that DDR2 was an important receptor for p38-activated osteoblast mineralization. We also found that the WRG-28 did not significantly change the gene expressions of *Ddr2*, this finding accords with its specific inhibition of receptor-ligand interaction through the receptor allosteric regulation [26]. An association between DDR2 and P38 during bone development has also been fully proven. DDR2 is a vital mediator of interactions between cells and fibrillar collagens [33]; it also stimulates osteoblast differentiation and bone formation signals through both ERK1/2 and p38 MAP kinase. These actions are highly related to changes in MAPK-dependent RUNX2 and PPARγ phosphorylation [24, 34]. Additionally, the positive role of P38 activation in the regulation of non-collagenous protein expression and osteoblast mineralization has also been confirmed in the previous studies [12]. In the third stage of the experiment, *Ddr2*-siRNA was constructed to transfect osteoblasts, and the mineralization indexes, including the protein determination of the mineralization index, ARS of mineralized nodules, and the ultrastructural observation of cells using TEM, were further determined. The results showed that the gene and protein expressions of DDR2 were decreased in siRNA group and the protein expression of P-DDR2 was decreased in inhibitor group; *Ddr2*-siRNA or DDR2 blockers also accordingly downregulated the expressions of non-collagen and P38 and decreased mineralization nodules; there were also fewer organelles and vesicles observed when compared with the ON group. The increase in osteoblast vesicles and organelles is an important sign of the enhancement of osteoblastic activity and mineralization ability. The disordered calcium phosphate in the vesicles is considered the precursor of carbonated hydroxyapatite, and mineral vesicles can initiate and directly participate in extracellular mineralization [35–37]. Mitochondria are involved in vesicle transport; amorphous calcium phosphate was found in mitochondria and new bone. There is also a strong point-to-point binding between mitochondria, intracellular calcium phosphate accumulation, and mineralization [38–40]. Combined, these results further clarified that both *Ddr2*-siRNA and DDR2 blockers could inhibit P38 activation and downregulate positive ON regulation of osteoblast mineralization.

In addition, P38 and mineralized gene expressions were still higher in both the inhibitor group and the *Ddr2*-siRNA group than in control group; the positive staining of calcium nodules areas were also larger. The presence of more than one activation pathway for P38 or other pathways by ON for regulating mineralization was considered. ON can activate P38 through several potential pathways, such as integrin [41, 42], matrix metalloproteinase [43–45], and transforming growth factor beta [46–48]. As ON is an extracellular matrix regulatory protein, its regulation of osteoblast mineralization is related to collagen synthesis regulation and the interaction between the extracellular matrix and osteoblasts. It was also found that the activity of osteoblasts was lower in the inhibitor group and the *Ddr2*-siRNA group than in other groups, indicating that DDR2 may be involved not only in mineralization but also in the entire process of osteoblast development and maturation. DDR2 is considered an important molecule for maintaining osteoblast activity and inhibiting bone marrow adipocyte formation [34]. Genetic evidence suggests that DDR2 plays a critical role in bone development. Furthermore, *Ddr2* mutations can cause spondylo-meta-epiphyseal dysplasia with short limbs and abnormal calcifications (SMED-SL) [49, 50], and *Ddr2* polymorphism is also strongly linked to a heightened fracture risk and lower bone mass measurements [51].

In summary, osteoblast mineralization is an extremely complex biological process, and several of its exact mechanisms still require clarification. Whether ON activates DDR2 via collagen alone or in other, more direct ways remains to be tested in future studies. Using the experiment conducted herein, it can be concluded that DDR2 is an important signaling pathway for ON activation of P38 in the regulation of osteoblast mineralization.

## Data Availability

We declared that materials described in the manuscript, including all relevant raw data, will be freely available to any scientist wishing to use them for non-commercial purposes, without breaching participant confidentiality.
